# Correlates of SuperAging in Two Population-Based Samples of Hispanic Older Adults

**DOI:** 10.1093/geroni/igaf122.1307

**Published:** 2025-12-31

**Authors:** Cassidy Doyle

**Affiliations:** University of South Florida, Pittsburgh, Pennsylvania, United States

## Abstract

“SuperAgers” are defined as people 80+ years old with episodic memory performance comparable to those 20 years younger. Limited knowledge exists to describe characteristics of SuperAgers, with even less known about Hispanic SuperAgers. We examined indicators of cognitive, physical, and psychological resilience in relation to the likelihood of being a SuperAger using data from 2 population-based studies of Hispanic older adults (Puerto Rican Elderly: Health Conditions [PREHCO] Study; Health and Retirement Study [HRS]). SuperAgers were defined as (1) ≥80 years old, (2) recall scores ≥ the median for Hispanic respondents aged 55–64, and (3) no cognitive impairment. Overall, 640 PREHCO participants and 180 HRS participants were eligible, of whom 45 (7%) and 31 (17%) met SuperAging criteria. Logistic regressions controlling for age and sex demonstrated that higher education (PREHCO: p < .001; HRS: p = .044) and fewer instrumental activities of daily living limitations (PREHCO: p = .019; HRS: p = .077; cognitive resilience), fewer activities of daily living limitations (PREHCO: p = .031; HRS: p = .068; physical resilience), and fewer depressive symptoms (PREHCO: p = .015; HRS: p = .007; psychological resilience) were associated with SuperAging, although not all results reached threshold for statistical significance. Known indicators of physical health (e.g., chronic conditions and self-rated health) did not relate to SuperAging. Increasing access to education and recognizing/treating depressive symptoms represent potential pathways to preserve episodic memory among older Hispanic adults.

